# Concurrent anemia and stunting in young children: prevalence, dietary and non-dietary associated factors

**DOI:** 10.1186/s12937-019-0436-4

**Published:** 2019-02-21

**Authors:** Shimels Hussien Mohammed, Bagher Larijani, Ahmad Esmaillzadeh

**Affiliations:** 1 Department of Community Nutrition, School of Nutritional Sciences and Dietetics, Tehran University of Medical Sciences-International Campus (TUMS-IC), Tehran, Iran; 2 Endocrinology and Metabolism Research Center, Endocrinology and Metabolism Clinical Sciences Institute, Tehran University of Medical Sciences, Tehran, Iran; 3 Obesity and Eating Habits Research Center, Endocrinology and Metabolism Molecular Cellular Sciences Institute, Tehran University of Medical Sciences, Tehran, Iran; 4 Department of Community Nutrition, School of Nutritional Sciences and Dietetics, Tehran University of Medical Sciences, Tehran, Iran; 5 Food Security Research Center, Department of Community Nutrition, Isfahan University of Medical Sciences, Isfahan, Iran

**Keywords:** Concurrent anemia and stunting, Anemia, Stunting

## Abstract

**Background:**

The existing evidence is limited and contradicting on the co-occurrence of anemia and stunting (CAS) at individual level, despite a great overlap in their risk factors. We aimed to determine the prevalence of CAS, and the dietary and non-dietary factors associated with it, among infants and young children in Ethiopia.

**Method:**

We used a nationally representative sample of 2902 children aged 6–23 months from the Ethiopian demographic and health survey, conducted in 2016. The study was cross-sectional in design. Samples were selected by two-stage clustering sampling method. CAS prevalence was estimated by various sociodemographic factors. To identify the dietary and non-dietary factors associated with CAS, we conducted hierarchical logistic regression analyses.

**Result:**

The overall prevalence of CAS was 23.9%. The dietary factors found significantly linked to lower odds of CAS were use of vitamin A supplement [adjusted odds ratio (AOR) = 1.19, 95%CI = 1.06–1.33, *P* = 0.003], consumption of vitamin A rich fruit and vegetables (AOR = 1.15, 95%CI = 1.04–1.27, *P* = 0.006), meat (AOR = 1.55, 95%CI = 1.17–2.05, *P* = 0.002), legumes (AOR = 1.38, 95%CI = 1.05–1.81, *P* = 0.021), and meal frequency > 3 (AOR = 1.22, 95%CI = 1.04–1.37, *P* = 0.020). The non-dietary household and child factors found significantly linked to higher odds of CAS were rural residence (AOR = 1.29, 95%CI = 1.18–1.41, *P* < 0.001), low household wealth (AOR = 1.91, 95%CI = 1.53–2.39, *P* < 0.001), low caregivers’ education level (AOR = 2.14, 95%CI = 1.33–3.44, *P* < 0.001), male sex (AOR = 1.25, 95%CI = 1.04–1.50, *P* = 0.015), age 12–23 months (AOR = 1.65, 95%CI = 1.57–1.73, *P* < 0.001), history of infection (AOR = 1.14, 95%CI = 1.00–1.30, *P* = 0.048), and small birth size (AOR = 1.99, 95%CI = 1.58–2.51, *P* < 0.001).

**Conclusion:**

Among infants and young children in Ethiopia, there was a concerning high level of CAS, which was associated with various dietary and non-dietary factors. Enhanced public health/nutrition interventions, with due emphasis on the multifactorial nature of CAS, might stand an important consideration to reduce the burden of CAS in Ethiopia and beyond.

## Introduction

Malnutrition remains a major public health challenge in Ethiopia, with anemia and stunting being the top two prevalent nutritional problems among infants and young children [1, 2]. The recent period has seen a significant and unexpected increment in anemia prevalence among under-5 Ethiopian children, rising from 44% in 2011 to 57% in 2016 [1]. According to the World Health Organization (WHO), anemia prevalence above 40% is classified as a severe public health problem and requires designing comprehensive interventions [3]. Stunting also remains a major problem despite it has been declining. In 2016, 38% of under-5 Ethiopian children were stunted [1].

Clustering of nutrition problems could occur at country, household, or individual levels. For most of the under-nutrition problems, there is a considerable risk factor overlap, particularly in the basic and underlying determinants [4–6]. Poor socioeconomic status, suboptimal child feeding and hygiene practices, and childhood illness coupled with poor health service utilization are often associated with multiple negative health outcomes, including malnutrition [5–8]. Thus, individuals are likely to be affected by double or even multiple forms of malnutrition. Anemia and stunting are of multiple overlapping influences originating from various levels [9–11]. Thus, a considerable co-occurrence of anemia and stunting (CAS) would be expected in settings with poor child care practices.

As each of anemia and stunting poses a significant challenge to the health system as well as the survival of children [12, 13], their co-occurrence (CAS) would be even more detrimental. However, studies on CAS are limited despite the availability of a large body of literature on each of anemia and stunting. Furthermore, the existing few studies on the clustering of the major forms of undernutrition, including CAS, are inconsistent [14–17]. Studies from Latin American countries reported a low level of anemia and stunting clustering. These studies recommended programmers to consider anemia and stunting as independent of each other and to focus on tailored, rather than comprehensive, malnutrition prevention interventions [15–17]. However, provided anemia and stunting share many of their basic and underlying risk factors, one could presume a child at risk of anemia be also at risk of stunting or vice versa. The existing national and international guidelines, including the WHO guidelines, also recommend adopting comprehensive and multi-sectoral malnutrition prevention strategies [12].

To the best of our knowledge, there is no previous study on CAS in Ethiopia as the existing studies were focused on either of anemia or stunting, i.e. did not report on CAS. In Ethiopia, both anemia and stunting are high. Child care and complementary feeding practices also remain poorly practiced [1]. Thus, we presumed there would be a high level of CAS for the two conditions share many risk factors. This study was aimed to determine the prevalence of CAS among infants and young children in Ethiopia and also investigate the dietary and non-dietary factors associated with it.

## Methods

### Data source

The data used for this work were extracted from the Ethiopian Demographic and Health Survey (EDHS). The survey was conducted in 2016 by the United States Agency for International Development (USAID) in collaboration with the Ministry of Health of Ethiopia and other partner organizations [1].

### Study design, sample size, and sampling procedures

**Fig. 1:**
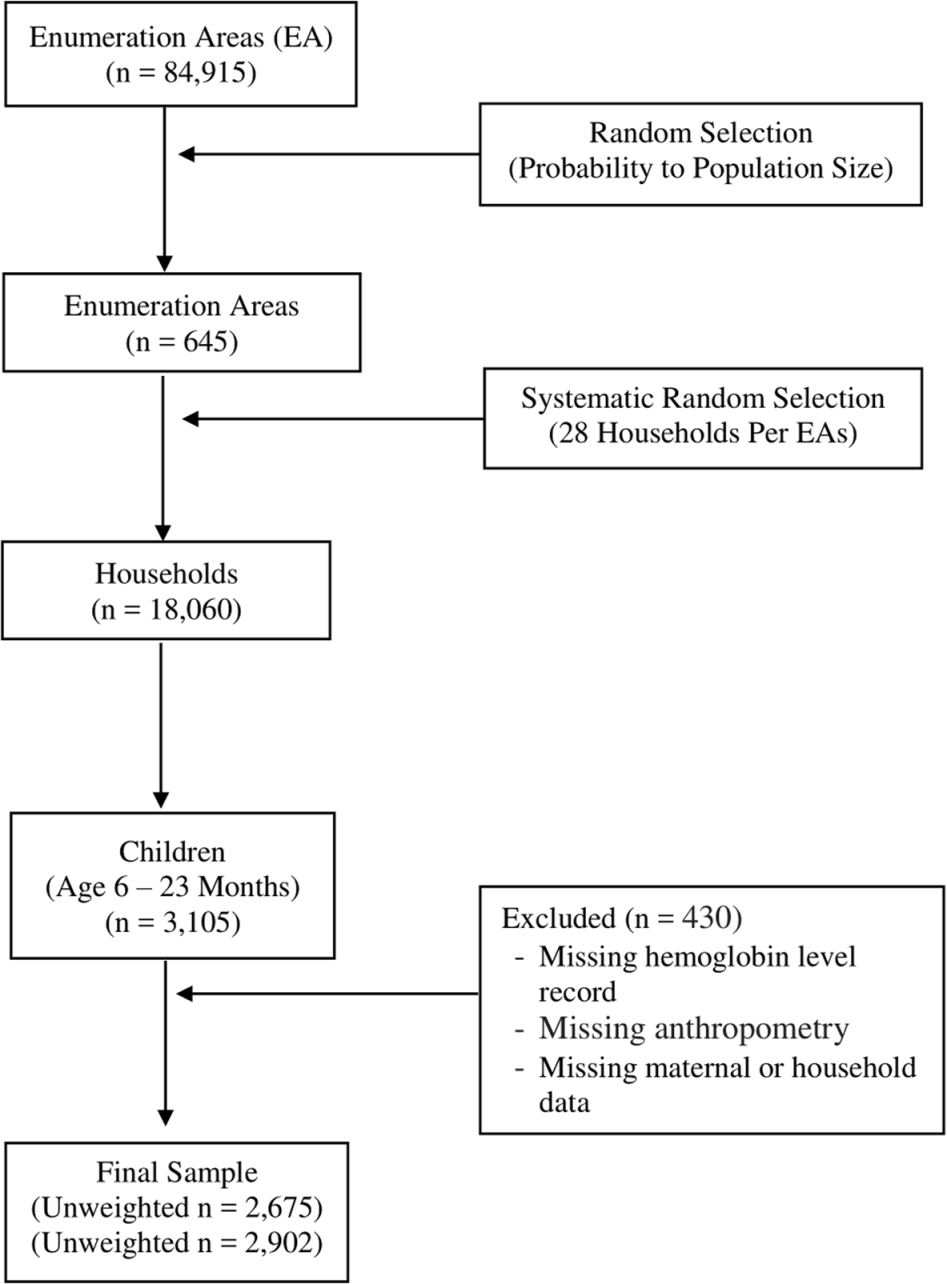
Flow chart of sample selection

The study was cross-sectional in design and representative at both national and regional levels, and urban and rural divisions [1]. The survey was done following a multistage sampling scheme. In the first stage of sampling, 645 enumeration areas (EAs) were randomly selected from all administrative divisions. The second sampling stage, selection of households within selected EAs, was done by systematic random sampling. In total, 18,060 households were selected. All children under-5 years of age in the selected households were eligible for the survey. More information about the EDHS sampling methodology and procedures is available elsewhere [1]. For this specific work, we extracted only the data of children below 24 months of age. Our interest in this age group was guided by two criteria: a) dietary and breastfeeding data were available only for the age group below 24 months, and b) most growth faltering (stunting) occurs during the first 2 years of life [11], during which the risk of anemia is also high. Infants below 6 months of age were also excluded because hemoglobin level was not measured for this age group during the conduction of the survey [1]. A total of 3105 children aged 6–23 months were found in the dataset. After data clearing and exclusion of cases with incomplete data on the variables of interest, 2675 children were included in the final dataset. Data incompleteness was only 13.8% (430 cases). The sample selection process is shown in Fig. 1.

### Data collection procedure

Data were taken on various demographic, health and nutrition variables of public health importance using the standardized DHS questionnaire, that has been used in Ethiopia as well as other countries included in the International DHS Project. The data collectors received a four weeks training on the survey questionnaire administration, anthropometric measurement, and biological sample collection procedures. Pilot testing and field practice were also done in clusters not included in the actual survey. The closest caregiver of the child, mostly the biological mother, was the primary source of information on the child-related variables of interest. When the biological mother could not be accessed, a member of the family responsible for the caring of the child was interviewed. The household head, mostly the father or the mother, was the primary informant on household-related variables of interest. All data were collected through house-to-house visits [1].

### Description of variables

The main variable of interest was CAS, defined when a child was both anemic and stunted. Anemia status was defined by hemoglobin < 11 g/dL [3]. Hemoglobin level was measured by battery operated HemoCue®201 analyzers (Sweden) and adjusted for altitude [1]. The length of children was measured in flat position using a Shorr measuring board, produced under the United Nations International Children’s Emergency Fund (UNICEF) guidance [1]. Length-for-age (LFA) z-scores were calculated using the WHO 2006 child growth standards. Stunting was defined by LFA < − 2 z-scores [18]. CAS prevalence, in this work, refers to the proportion of children with both anemia and stunting. A list of the potential predictors of CAS was developed, guided by the literature, the UNICEF conceptual framework of malnutrition causation [12], and the availability of the variables in the dataset. Based on their level of influence and interrelationships, the variables were categorized into three groups: basic, underlying, and proximal factors. A detailed description of the explanatory variables is presented in Table 1.

**Table 1:** Description of variables

Variables	Description
Concurrent anemia and stunting (CAS)	Defined when a child was both anemic and stunted. Anemia status was measured by hemoglobin level < 11 g/dL and categorized as anemic and non-anemic [3]. Stunting was measured by length-for-age < − 2 z-score and categorized as stunted and non-stunted.
*Basic (distal) factors*	
Residence place	Categorized as urban and rural.
Region	Categorized as mainly agrarian and mainly pastoral.
Household wealth category	First, household wealth index was developed by principal component analysis, using asset variables collected during the survey [19]. Then, the index was used to rank the households into low, middle and high wealth categories.
Caregiver’s education status	Categorized as illiterate/none, primary, and secondary and above.
*Underlying (intermediate) factors*	
Water source	Categorized as improved and unimproved.
Toilet facility	Categorized as improved and unimproved.
*Proximal (immediate) factors*	
Child sex	Categorized as boy and girl.
Child age	Categorized into < 12 and 12–23 months of age.
Birth size	Assessed by the subjective reporting of the mother about the size of the child at birth and categorized as large, average and small. Birth size was used as a proxy measure of birth weight.
History of infection	Assessed by whether the child had fever, diarrhea, or cough in the last two weeks preceding the survey. Categorized as yes and no.
Breastfeeding duration	Categorized as < 12 and 12–23 months.
Early breastfeeding initiation	Assessed by initiation of breastfeeding within the first hour after birth. Categorized as yes and no.
Deworming tablet use	Assessed by whether the child received deworming table within the previous 6 months. Categorized as yes and no.
Vitamin A supplement use	Assessed by whether the child received vitamin A supplement within in the previous 6 months. Categorized as yes and no.
Iron supplement use	Assessed by whether the child received iron supplement within the previous 7 days. Categorized as yes and no.
Complementary food groups	During the survey, caregivers were asked to recall the type of food the child was fed in the 24 h (one day) prior to the survey. The frequency of feeding during the same period was also assessed. The data were collected by trained interviewers, following the 5-Step Multiple-Pass Method. Using the WHO/UNICEF [39] and the DHS guidelines [19] for the assessment of infant and young child feeding practices, the food items were further categorized into 7 food groups: (1) meat, (2) egg, (3) milk, (4) grains, roots, and tubers, (5) legumes and nuts, (6) vitamin-A rich fruits and vegetables (VARFV), and (7) other fruits and vegetables (OFV). Using the 7 food groups above and following the WHO/UNICEF [39] and the DHS guidelines [19], dietary diversity and meal frequency indexes were determined for each child. The diversity and frequency indexes were used to determine whether the child met the WHO/UNICEF recommendations for dietary diversity and meal frequency.
Minimum dietary diversity (MDD)	Assessed by whether the child received ≥4 complementary food items from the 7 food groups within the previous 24 h. Categorized as yes and no [39].
Minimum meal frequency (MMF)	Assessed by whether the child was fed with complementary food ≥3 times if on breastfeeding, or ≥ 4 times if not on breastfeeding, within the previous 24 h. Categorized as yes and no [39].

### Statistical analysis

All analyses were done taking into account the complex design of the survey, such that all the estimates were done after weighting the sample by the sample weighting factor and taking into account the cluster design of the study. While it ensures data representativeness at both national and subnational levels, the DHS sampling procedure over-represents small regions. Thus, sample weighting was applied to compensate for the unequal probability of sample selection and ensure the sample resembles the true population distribution. The procedure resulted in a final weighted sample size of 2902 children. Besides, as the study participants were selected by two-stage cluster sampling strategy, adjustment for effect of the cluster design was done by applying the STATA command ‘svyset:’. More information about the DHS sample weighting and adjustment for cluster design is found in the guide to DHS statistics [19].

CAS prevalence by various characteristics was estimated. Adjusted odds ratios (AOR) were estimated by running multiple hierarchical logistic regression analyses, which took into account the hierarchical interrelationship among the potential predictors of CAS. The selection of the variables was done taking into account both biological (theoretical) plausibility and statistical assumptions for multivariate analyses. Thus, bivariate analyses were done for all variables with the potential to influence CAS. Then, multivariate models were run using variables which demonstrated *P* < 0.20 during the bivariate analyses. The reasons for our use of a relaxed *P*-value (*P* < 0.20), instead of the usual *P* = 0.05, were: a) the purpose of the bivariate analyses was to identify potential predictor variables for the multivariate analyses rather than testing hypothesis, and b) it would minimize the risk of excluding variables with a biological (theoretical) plausibility from the multivariate analyses due to reasons, including confounding [20, 21]. However, the statistical significance of the results of the multivariate analyses was determined at *P* ≤ 0.05, because of its common usage in medical research.

After the bivariate analyses, three-level hierarchical regression models were run following the recommendation of Victoria et al. [22]. The first, second, and third models contained distal, underlying, and proximal factors, respectively. Variables with *P* < 0.20 value during the bivariate analyses were included in the multiple hierarchical logistic regression analyses. Thus, Model-1 included the distal factors which demonstrated *P* < 0.20 during the bivariate analyses. Model-2 included the underlying factors which demonstrated *P* < 0.20 during the bivariate analyses and factors from model-1 which demonstrated *P* < 0.20. Model-3 included the proximal factors and factors from Model-2 which demonstrated *P* < 0.20. Statistical significance (*P* ≤ 0.05) of a specific variable during the hierarchical regression analyses was determined at the corresponding model in which the variable was first entered, irrespective its performance in the next model(s). The approach was aimed to rule out the possibility that the intermediate factors weaken the relationship between the distal factors and the dependent variable of interest [22]. All statistical analyses were done using STATA-15.

## Result

### Sociodemographic characteristics of the sample

The main characteristics of the study participants are shown in Table 2. In totally, the study included 2902 (weighted) children aged 6 to 23 months, a third of whom were younger than 12 months. Mean age was 14.0 months (SD = 5.1). 1542 (53.1%) were girls and 1360 (46.9%) boys. Most of the participants were from rural areas (88.1%) and of less educated caregivers.

**Table 2:** Bivariate analysis of the relation of basic, underlying, and proximal factors with CAS (Weighted *N* = 2902)

Variables		Weighted frequency (%)	CAS Prevalence (95% CI)	*P*-value*
*Basic factors*				
Residence place	Urban	11.9	16.1 (12.0–20.2)	0.001
	Rural	88.1	25.0 (23.3–26.7)	
Region (state)	Pastoral	6.4	22.6 (16.2–29.1)	0.606
	Agrarian	93.6	24.4 (22.8–26.1)	
Wealth category	Low	44.1	31.7 (26.4–37.1)	< 0.001
	Middle	22.5	16.4 (11.0–21.9)	
	High	33.4	14.5 (11.0–17.9)	
Caregivers education	No	61.0	23.4 (19.6–27.2)	< 0.001
	Primary	30.6	18.5 (14.1–22.9)	
	Secondary+	8.4	13.5 (6.4–20.5)	
*Underlying factors*				
Water source type	Not improved	50.1	22.9 (19.1–26.8)	0.065
	Improved	49.9	17.9 (14.1–21.7)	
Toilet facility type	Not improved	90.2	21.1 (18.2–24.1)	0.001
	Improved	9.8	17.2 (10.3–24.1)	
*Proximal factors*				
Child sex	Boy	46.9	21.2 (17.2–25.2)	0.033
	Girl	53.1	20.1 (16.5–23.8)	
Child age	< 12 months	34.9	12.1 (8.5–15.7)	< 0.001
	12–23 months	65.1	25.4 (21.8–29.1)	
Birth size	Small	27.0	29.7 (23.7–35.8)	< 0.001
	Average	40.7	19.1 (15.1–23.2)	
	Large	32.3	15.3 (11.0–19.5)	
Infection history^a^	No	62.4	20.0 (16.6–23.4)	0.031
	Yes	37.6	21.6 (17.2–25.9)	
Breastfeeding duration	< 12 months	38.4	22.1 (18.5–25.7)	0.692
	12–23 monhs	61.6	25.4 (21.8–29.1)	
Early breastfeeding initiation	No	10.9	22.4 (14.0–30.9)	0.533
	Yes	89.1	20.4 (17.6–23.2)	
Deworming	No	90.8	20.5 (17.7–23.3)	0.802
	Yes	9.2	22.0 (12.2–31.8)	
Vitamin A supplement	No	56.4	18.0 (14.5–21.5)	0.043
	Yes	43.6	23.8 (19.6–28.0)	
Iron supplement	No	92.1	20.1 (17.2–22.9)	0.337
	Yes	7.9	25.4 (16.4–34.5)	
Grains and tubers	No	27.8	25.5 (19.6–31.4)	0.349
	Yes	72.2	19.1 (16.1–22.1)	
VARFV	No	72.3	21.3 (18.1–24.5)	0.088
	Yes	27.7	18.9 (14.0–23.8)	
OFV	No	82.8	22.2 (19.2–25.3)	0.006
	Yes	17.2	14.1 (8.9–19.3)	
Meat	No	91.6	21.5 (18.6–24.3)	0.050
	Yes	8.4	13.8 (6.8–20.9)	
Milk	No	61.7	22.2 (18.7–25.6)	0.013
	Yes	38.3	18.0 (13.7–22.2)	
Legumes and nuts	No	79.1	22.3 (19.1–25.4)	0.064
	Yes	20.9	14.8 (9.7–19.9)	
Egg	No	83.3	21.3 (18.3–24.3)	0.348
	Yes	16.7	17.9 (12.0–23.7)	
MDD^b^	No	84.6	22.7 (19.7–25.8)	0.010
	Yes	15.4	11.0 (0.06–15.9)	
MMF^c^	No	57.3	19.4 (15.9–22.9)	0.022
	Yes	42.7	22.3 (18.0–26.5)	

CAS: concurrent anemia and stunting, CI: confidence interval, VARFV: vitamin A rich fruits and vegetables, OFV: other fruits and vegetables

^a^ = Infection defined as history of cough, diarrhea, or fever in the last 2 weeks (yes, any one of the three conditions)

^b^ = Minimum dietary diversity defined as, according to the WHO criteria, eating from 4 or more of the 7 food groups

^c^ = Minimum meal frequency defined as, according to the WHO criteria, when a child ate at least 3 and 4 times a day for breastfeeding and non-breastfeeding, respectively

^*^Based on Chi-square test of association

### Prevalence of CAS

Overall, 72 and 32.6% of children were anemic and stunted, respectively. The overall prevalence of CAS was 23.9%. CAS prevalence among urban and rural children was 16.1 and 25.0%, respectively. Among boys and girls, the prevalence was 21.2 and 20.1%, respectively. The age-specific estimates were 12.1 and 25.4% in those aged under 12 months and above 12 months, respectively. The prevalence of CAS by other maternal and child characteristics is shown in Table 2.

### Factors associated with CAS

Results of the bivariate analyses of the basic, underlying, and proximal variables with CAS are shown in Table 2. These estimates were, however, crude and less informative, i.e. not adjusted for any covariate. The co-variate adjusted estimates, i.e. based on the hierarchical regression analyses, are presented in Table 3. Rural residence was associated with a significantly higher odds of CAS (AOR = 1.29, 95%CI = 1.18–1.41, *P* < 0.001), compared to urban residence. The odds of CAS was significantly higher in children of low household wealth category, compared to children of high household wealth category (AOR = 1.91, 95%CI = 1.53–2.39, *P* < 0.01). Compared to children of caregivers with secondary+ education level, the odds of CAS was 2.14 times higher in children of caregivers with no education (95%CI = 1.33–3.44, *P* < 0.01) and 2.38 times higher in children of caregivers with only primary education (95%CI = 1.47–3.86, *P* < 0.01). In boys, the likelihood of CAS was 1.25 times that of girls (95%CI = 1.04–1.50, *P* = 0.015). Above 12 months of age was significantly associated with a higher odds of CAS, compared to below 12 months of age (AOR = 1.65, 95%CI = 1.57–1.73, *P* < 0.001). Small birth size was significantly associated with a higher odds of CAS, compared to large birth size (AOR = 1.99, 95%CI = 1.58–2.51, *P* < 0.001). Infection was linked to a marginally higher odds of CAS (AOR = 1.14, 95%CI = 1.00–1.30, *P* = 0.048).

**Table 3:** Hierarchical multiple logistic regression analysis of the relation of basic, underlying, and proximal factors with CAS

Variables		Model-1^a^		Model-2^b^		Model-3^c^	
		AOR (95%CI)	P*	AOR (95%CI)	P*	AOR (95%CI)	P*
Residence place	Urban	Reference	< 0.001				
	Rural	1.29 (1.18–1.41)					
Household wealth category	Low	1.91 (1.53–2.39)	< 0.001				
	Middle	1.10 (0.84–1.44)	0.480				
	High	Reference					
Caregiver education	No education	2.14 (1.33–3.44)	< 0.001				
	Primary	2.38 (1.47–3.86)	< 0.001				
	Secondary+	Reference					
Water source	Unimproved			1.08 (0.81–1.45)	0.606		
	Improved			Reference			
Toilet facility	Unimproved			0.96 (0.58–1.57)	0.857		
	Improved			Reference			
Child sex	Girl					Reference	0.015
	Boy					1.25 (1.04–1.50)	
Child age	< 12 months					Reference	< 0.001
	12–23 months					1.65 (1.57–1.73)	
Birth size	Large					Reference	
	Average					1.21 (0.90–1.40)	0.317
	Small					1.99 (1.58–2.51)	< 0.001
Infection^d^	No					Reference	0.048
	Yes					1.14 (1.00–1.30)	
Vitamin A supplement	No					1.19 (1.06–1.33)	0.003
	Yes					Reference	
VARFV	No					1.15 (1.04–1.27)	0.006
	Yes					Reference	
OFV	No					1.25 (0.90–1.73)	0.178
	Yes					Reference	
Meat	No					1.55 (1.17–2.05)	0.002
	Yes					Reference	
Milk	No					1.14 (0.90–1.44)	0.276
	Yes					Reference	
Legumes	No					1.38 (1.05–1.81)	0.021
	Yes					Reference	
MDD^e^	No					1.29 (0.93–1.78)	0.122
	Yes					Reference	
MMF^f^	No					1.22 (1.04–1.37)	0.020
	Yes					Reference	

*CAS* concurrent anemia and stunting, *AOR* adjusted odds ratio, *CI* confidence interval, *VARFV* vitamin A rich fruits and vegetables, *OFV* other fruits and vegetables

^a^Model-1: adjusted for residence place, wealth category and caregiver’s education status

^b^Model-2: adjusted for residence place, wealth category, caregiver’s education and all variables shown under Model-2

^c^Model-3: adjusted for residence place, wealth category, caregiver’s education, and all variables shown under Model-3

^d^ = Infection defined by history of cough, diarrhea, or fever in the last 2 weeks (yes, any one of the three conditions)

^e^ = Minimum dietary diversity defined as, according to the WHO criteria, eating from 4 or more of the 7 food groups

^f^ = Minimum meal frequency defined as, according to the WHO criteria, when a child ate at least 3 and 4 times a day for breastfeeding and none-breastfeeding, respectively

*P-value significant when < 0.05

Children who did not take vitamin A supplement within the previous six months had a higher odds of CAS, compared to those who took (AOR = 1.19, 95%CI = 1.06–1.1.33, *P* = 0.003). The odds of CAS was also significantly higher in children who did not receive VARFV, compared to those who received (AOR = 1.15, 95%CI = 1.04–1.27, *P* = 0.006). The odds of CAS related to no meat consumption was 1.55 times higher compared to its consumption (95%CI = 1.17–2.05, *P* = 0.002). The odds of CAS related with no legumes consumption was 1.38 times, higher compared to its consumption (95%CI = 1.05–1.81, *P* = 0.021). Meal frequency was also significantly associated with CAS, such that the odds of CAS was 1.22 times higher in those who didn’t meet MMF, compared to those who meet it (95%CI = 1.04–1.37, *P* = 0.020).

## Discussion

This was the first study aimed to determine the prevalence of CAS, and its multilevel associated factors, among infants and young children in Ethiopia. The work provided evidence that there was a high level of anemia and stunting co-occurrence in Ethiopia, with almost a quarter of the children affected. We found CAS linked to various multiple-level influences. The distal factors associated with higher odds of CAS were living in rural areas, low household wealth, and low caregivers’ educational level. The proximal factors found associated with higher odds of CAS were male sex, age above 12 months, small birth size, no vitamin A supplement use, no consumption of vitamin A rich food items, meat and legumes, and low meal frequency.

We found a concerning high level of anemia, with only less than a third of the children being not anemic. The finding was, however, consistent with the recent national DHS report which showed 72 and 56% anemia prevalence among under-2 and under-5 children, respectively [1]. Stunting was also highly prevalent, though not as high as anemia. The same finding was reported in previous studies, which showed a two-fifth prevalence of stunting among under-5 Ethiopian children [1, 13, 23]. We found that almost a quarter of the children were concurrently anemic and stunted. There was no previous report on the magnitude of CAS, and factors associated with it, in Ethiopia as well as other African countries, limiting the comparison of our findings. However, our finding was within the range of reports from Asian and Latin American countries. Albalak et al. [17] reported a 15.2% CAS prevalence in Honduras. Castejon et al. [15] reported a 5.9% CAS prevalence in Venezuela. CAS prevalence was 21.5% in India and 30.4% in Peru [16].

We found various factors linked to CAS. Children of low household wealth or caregivers of low education level were more likely to be concurrently anemic and stunted. These findings could be easily acknowledged because child health-enhancing behaviors, like proper feeding, hygiene, and utilization of health services, are often sub-optimally practiced among communities of low wealth and educational status [8, 11, 24]. We also found a more clustering of CAS in those above 12 months of age. This could be due to the nature of stunting that it takes more time to manifest than anemia which takes a shorter time. The existing literature shows that most stunting occurs more during the period 12 to 23 months of age [11, 25]. In general, children under-2 years of age bear a higher burden of both anemia and stunting, particularly in developing countries [4, 9, 12]. Our finding of higher odds of CAS in boys than in girls was in agreement with previous reports which consistently demonstrated higher risks of anemia and stunting in boys [10, 25, 26]. Small birth size was associated with a significantly higher odds of CAS. This finding was also consistent with the existing literature which shows low birthweight linked to various poor health and nutritional outcomes [25, 26].

Vitamin A intake, in dietary as well as supplement form, was associated with a significantly lower CAS prevalence. This would be most likely due to the role of vitamin A in promoting optimal hematologic and linear growth status [24, 27–29]. Vitamin A also boosts humoral as well as cell-mediated immunity, thereby reducing the risk of anemia due to infection [29]. Vitamin A also plays an important role in promoting child growth, thereby reducing the risk of stunting [27, 28, 30]. Thus, it could be easily acknowledged that a vitamin A deficient child would be at a higher risk of being concurrently affected by anemia and stunting. Lack of meat and legumes consumption, as well as low meal frequency, were independently associated with higher odds of CAS. This could be due to the better amino acid and iron profiles in meat and legumes [31, 32]. Thus, suboptimal intake of legume or meat products might be expected to negatively impact both hemoglobin and growth statuses [23, 31].

Among the dietary factors, milk consumption was not significantly linked to CAS. This could be, in part, because most of the study participants were of less educated caregivers and from rural areas, where animal milk is consumed mainly raw. Previous reports showed opposing effects of raw animal milk on height and hemoglobin statuses [33, 34]. It promotes height gain, thus reduces the risk of stunting [34, 35], but predisposes to gastroenteritis and occult bleeding, thus increases the risk of anemia [33, 36]. Iron supplement use also did not demonstrate a significant link to CAS, though it would be expected to promote both hemoglobin and height [7]. Our finding could be likely due to factors like: a) the children taking iron supplement might be the already anemic ones, b) we did not account for dose, frequency, and adherence to the iron supplement use, or c) the sample lacked the power to detect the association, if any, because the number of children who took iron supplement was low. Notwithstanding the role of iron in red blood cells formation and body growth, previous randomized control and meta-analysis studies also reported as iron supplement use lacked a demonstrable effect on physical growth as well as hemoglobin level of children [37, 38].

The findings of this study have important policy and research implications. The high level of CAS was concerning given each of the two conditions are of significant consequences and their co-occurrence would be more threating to the health of children. It is also important to note the criticality of the first 1000 days of life, during which the body is more vulnerable to both nutritional and non-nutritional threats [4, 11]. Thus, the finding might be an indicator of the need to report on CAS and also investigate whether these children are being reached with a priority through the existing health/nutrition programs. Currently, there is confusion on how to address the various forms of malnutrition. To address stunting and anemia, WHO guidelines recommend a comprehensive and integrated approach, as it also has multiple benefits [12, 24]. Some authors, however, questioned the approach arguing that anemia and stunting are independent of each other and better be addressed by tailored interventions [15–17]. We are of the WHO recommendation as anemia and stunting share most of their risk factors. Thus, we recommend strengthening the existing public health and nutrition efforts, including improving infant and young child feeding practices, micronutrient supplementation, hygiene, and health care.

The main strengths of this study were it was based on a nationally representative data and took into account the multi-factorial nature of CAS, by incorporating not only the immediate dietary factors but also the non-dietary factors with the potential to influence CAS. Our analysis approach, hierarchical regression, took into account the interrelationships among the various explanatory variables and enabled building models according to the level of the variable influence. One of the main limitations of the study was that a cause-effect relationship could not be inferred as it was based on cross-sectional data. The collection of data on some variables, like birth size, infection history, dietary frequency, and diversity, based on the subjective memory of the caregiver might have introduced recall bias and miss-classification.

## Conclusion

In conclusion, we provided evidence that there was a concerning high level of anemia and stunting clustering among infants and young children in Ethiopia. CAS was associated with various dietary and non-dietary factors, originating from community, maternal and child levels. Strengthening the existing comprehensive public health/nutrition interventions, with due emphasis on the multifactorial nature of CAS, might stand an important consideration to reduce the burden of CAS in Ethiopia and beyond.

## Abbreviations

AOB: adjusted odds ratio
CAS: concurrent anemia and stunting
CI: confidence interval
EDHS: Ethiopian demographic health survey
MDD: minimum dietary diversity
MMF: minimum meal frequency
SD: standard deviation
UNICEF: United Nations International Children’s Emergency Fund
VARFV: vitamin A rich fruit and vegetables
WHO: World Health Organization
